# Somatostatin binding in normal and malignant human gastrointestinal mucosa.

**DOI:** 10.1038/bjc.1992.275

**Published:** 1992-08

**Authors:** G. V. Miller, S. M. Farmery, L. F. Woodhouse, J. N. Primrose

**Affiliations:** University Department of Surgery, St. James's University Hospital, Leeds, UK.

## Abstract

Somatostatin is a regulatory peptide implicated in the control of cellular proliferation in epithelial tissues and this regulation may occur directly via membrane bound receptor activation. The aim of this study was to investigate somatostatin binding in human gastrointestinal cancer and normal mucosa. Plasma membranes were prepared from specimens of tumour and normal mucosa from 51 patients undergoing surgical resection for malignancy (28 gastric, 23 colorectal). Using a competitive displacement assay, specific 125I-tyrosine-11-somatostatin-14 binding to plasma membranes was assessed and and characterised in terms of receptor affinity (Kd) and maximum binding capacity (Bmax) as determined by Scatchard analysis. Specific low affinity (Kd = 166 nM), high capacity (Bmax = 1.2 pmol mg-1 protein) somatostatin binding was demonstrated in 22 of the gastric cancers and 17 of the colorectal cancers (Kd = 140 nM, Bmax = 1.8 pmol mg-1 protein). Similar affinity and binding capacity was demonstrable in normal mucosal samples. High affinity receptors for somatostatin were expressed by one gastric carcinoma (Kd = 0.9 nM; Bmax = 0.23 pmol mg-1 protein). Thus, low affinity, high capacity binding is a common feature of gastrointestinal tumours and normal mucosa, and high affinity receptors may occasionally be demonstrated. The functional significance of these low affinity binding sites requires elucidation to determine whether long-acting somatostatin analogues may have therapeutic benefit in gastrointestinal malignancy.


Br. J. Cancer (1992), 66, 391-395 © Macmillan Press Ltd., 1992

### Somatostatin binding in normal and malignant human gastrointestinal mucosa

G.V. Miller, S.M. Farmery, L.F. Woodhouse & J.N. Primrose

*University Department of Surgery, St. James's University Hospital, Beckett Street, Leeds LS9 7TF, UK.*

**Summary** Somatostatin is a regulatory peptide implicated in the control of cellular proliferation in epithelial tissues and this regulation may occur directly via membrane bound receptor activation. The aim of this study was to investigate somatostatin binding in human gastrointestinal cancer and normal mucosa. Plasma membranes were prepared from specimens of tumour and normal mucosa from 51 patients undergoing surgical resection for malignancy (28 gastric, 23 colorectal). Using a competitive displacement assay, specific $^{125}$I-tyrosine -11-somatostatin-14 binding to plasma membranes was assessed and characterised in terms of receptor affinity (Kd) and maximum binding capacity (Bmax) as determined by Scatchard analysis. Specific low affinity (Kd = 166 nM), high capacity (Bmax = 1.2 pmol mg$^{-1}$ protein) somatostatin binding was demonstrated in 22 of the gastric cancers and 17 of the colorectal cancers (Kd = 140 nM, Bmax = 1.8 pmol mg$^{-1}$ protein). Similar affinity and binding capacity was demonstrable in normal mucosal samples. High affinity receptors for somatostatin were expressed by one gastric carcinoma (Kd = 0.9 nM; Bmax = 0.23 pmol mg$^{-1}$ protein). Thus, low affinity, high capacity binding is a common feature of gastrointestinal tumours and normal mucosa, and high affinity receptors may occasionally be demonstrated. The functional significance of these low affinity binding sites requires elucidation to determine whether long-acting somatostatin analogues may have therapeutic benefit in gastrointestinal malignancy.

Hormonal manipulation, by surgical or medical methods, has been used extensively in therapy for carcinoma of the breast, prostate, thyroid and in certain gut endocrine tumours (Horowitz *et al.*, 1975; Higgins *et al.*, 1941; Crile, 1957; Osei *et al.*, 1985). There is, however, no currently defined role for endocrine therapy in non-endocrine gastrointestinal cancer. Surgical intervention is the most effective treatment for this condition and, even then, can only afford a 'cure' in early disease. Advanced tumours are associated with a poor prognosis, marginally improved by chemotherapeutic regimens. Any benefit from systemic therapy is usually abrogated by systemic toxicity (Rake *et al.*, 1979; Engstrom *et al.*, 1985; Bleiberg, 1990). For this reason a non-toxic, effective, treatment modality would be of value to improve the outlook for those patients with advanced disease.

There is increasing evidence that tumours arising from the gastrointestinal tract are, at least in part, hormone dependent. Numerous hormones have now been implicated in the pathogenesis and development of gastrointestinal malignancy, including gastrin, epidermal growth factor, enteropancreatic hormones and oestrogenic steroids (Sirinek *et al.*, 1985; Watson *et al.*, 1988; Li *et al.*, 1980; Howatson & Carter, 1985; McMichael *et al.*, 1980). These findings, together with the observation that transformed gut epithelial cells may retain functional hormone receptors (Townsend *et al.*, 1986), have suggested a role for hormonal manipulation in these malignancies.

Somatostatin and its analogues are good candidates for use as endocrine agents in the treatment of gastrointestinal cancer. The native peptide is widely distributed in the body and, amongst its many inhibitory actions, has a putative role as an anti-proliferative agent in both normal (Lehy *et al.*, 1979) and malignant (Singh *et al.*, 1986) tissue. The mechanisms involved in this anti-proliferative action have not, as yet, been confirmed. Both the native peptide and its analogues act at the somatotrophs of the anterior pituitary suppressing growth hormone production and release (Adrian *et al.*, 1981). This may influence the proliferation of target tissues directly or may result in a substantial reduction in local growth factor release (Kirkegaard *et al.*, 1984). Further, the production of other trophic hormones such as gastrin and insulin, either from normal cells or from hormonally active tumour cells, could be suppressed by somatostatin (Morris *et al.*, 1988). The most interesting mechanism, however, involves the interaction of the peptide with specific membrane bound receptors upon proliferating cells. Certain tumours are known to express somatostatin receptors, and these include meningioma, carcinoid and related gut endocrine tumours, small cell carcinoma of the lung and growth hormone-secreting pituitary adenomas (Reubi *et al.*, 1987*a*; Reubi *et al.*, 1987*b*; Reubi *et al.*, 1990; Ikuyama *et al.*, 1985).

Somatostatin binding has not been investigated in human gastrointestinal cancer. The aim of this study, therefore, was to determine whether there was specific binding of somatostatin to malignant gastrointestinal tissue and adjacent uninvolved mucosa, and to characterise any such binding. This information may help determine whether somatostatin and its analogues have a role in gastrointestinal cancer.

### Patients, materials and methods

### *Collection and storage of normal and malignant tissue*

Tumour tissue and uninvolved mucosa were obtained at operation from 51 consecutive patients (26 male, 25 female; mean age (range) 70.4 (42-86) years) with gastrointestinal cancer. Twenty eight of these patients suffered from gastric cancer and the remaining 23 were operated upon for colonic or rectal cancer. Resected specimens were opened and washed in cold 0.9% saline immediately after surgical removal. Incisional biopsies of macroscopically viable tumour were removed and adjacent blocks processed for routine histopathology. Macroscopically normal mucosa from a site distant from the tumour was carefully dissected from the underlying muscular layers. Tumour and normal mucosal samples were finely divided, snap frozen in liquid nitrogen and stored at −70°C until assayed.

### *Preparation of plasma membranes*

Plasma membranes were prepared using a modification of the method of Srikant and Patel (1981). In brief, frozen tumour and mucosal samples were mechanically pulverised and homogenised on ice in homogenising buffer (sucrose 250 mM, KCl 25 mM and $MgCl_2$ 10 mM in Tris-HCl 50 mM; pH 7.4) at 10,000 r.p.m. in short bursts for 2 min using an Ultraturrax T25 homogeniser (Scientific Instruments Ltd, Liverpool,

Correspondence: J.N. Primrose, University Department of Surgery, Clinical Sciences Building, St James's University Hospital, Beckett St, Leeds LS9 7TF, UK.
Received 18 November 1991; and in revised form 9 March 1992.

UK). The homogenate was centrifuged at 4°C at 270 g for 10 min to remove nuclear debris and the supernatant retained. This crude membrane suspension was then ultracentrifuged at 4°C for 10 min at 15,000 g using a Beckmann L5 65B Ultracentrifuge (Beckmann Laboratory Instruments, High Wycombe, Bucks, UK). The pellet was resuspended in Tris-HCl buffer (Tris-HCl 10 mM, pH 7.4) and ultracentrifuged for 10 min at 15,000 g. The final pellet was resuspended in 2 ml of the Tris-HCl buffer, pH 7.4, and the protein content determined after the method of Bradford (1976).

Rat cerebral cortex was used as a positive control for all binding assays. Six week old female Wistar rats were sacrificed by cervical dislocation and the cerebral hemispheres dissected from the remaining cranial contents. Plasma membranes were prepared as outlined above and used at a final protein concentration of 0.2 mg ml$^{-1}$.

*Preparation of $^{125}$I-tyr-11-somatostatin-14*

Iodination of tyr-11-somatostatin-14 (Sigma Ltd, UK) was performed using the Chloramine T method (Czernick & Petrack, 1983). The iodination reaction mixture was eluted on a G-25 Superfine Sephadex column using an elution buffer of 0.1 M acetic acid containing 1 mg ml$^{-1}$ bovine serum albumin. Eluted fractions were collected and the specific activity of the combined $^{125}$I-tyr-11-somatostatin-14 peak was calculated to be between 330 and 500 Ci mmol$^{-1}$.

*Demonstration of somatostatin binding*

A twelve point competitive displacement assay was developed and validated using plasma membranes prepared from rat cerebral cortex. Incubation buffer (HEPES[3]-KOH 50 mM, $MgCl_2$ 10 mM, BSA 1% and bacitracin 0.01%; pH 7.4), 50 µl, was added to each reaction tube. Unlabelled somatostatin-14 (Sigma Ltd, UK) was then added in 10 µl aliquots over the concentration range $10^{-11}$ M to $10^{-5}$ M. Total binding was assessed by the addition of 10 µl of 50 mM HEPES-KOH pH 7.4. An aliquot of 20 µl of plasma membrane preparation at a protein concentration of 1–1.5 mg ml$^{-1}$ was added to the reaction tubes. Finally 20 µl (100,000 c.p.m.) of radioisotope (approx 2 nM) was added to each tube. Samples were incubated for 1 h at 35°C, the reaction being stopped by the addition of 0.5 ml ice cold saline, and centrifugation at 13,000 g for 2 min in a Microcentaur microcentrifuge (MSE Ltd, UK). The supernatant was removed by suction, the pellet washed once with 0.5 ml ice cold saline and counted in the reaction tube on a Packard Cobra II Autogammacounter (Canberra Packard, Pangbourne, Berks, UK). All assay points were performed in triplicate and the coefficient of variation of the triplicates was less than 10%.

*Tumour site, stage and histological grade*

The tumour site, stage and histological grade were assessed by routine pathological methods by one histopathologist. The degree of differentiation (Well, Moderate or Poor) of the tumour was determined from the block of tissue adjacent to that used in the binding studies. Staging of colonic/rectal carcinoma was performed according to Dukes' classification into stage A, B or C. Gastric adenocarcinoma was staged according to the TNM classification and patients allocated to Stage I–IV (after Fielding *et al.*, 1983).

*Data analysis and statistical methods*

Binding data was processed using the Ligand PC Curve Fitting Program (Munson & Rodbard, 1980) to derive receptor affinity, Kd (nM) and maximum binding capacity (receptor density), Bmax, expressed in terms for the protein concentration of the membrane preparation. Data was analysed using non parametric statistical tests of unpaired data and all values are expressed as median (quartiles) unless otherwise stated.

### Results

*Somatostatin binding in rat cerebral cortex*

Binding of $^{125}$I-tyr-11-somatostatin-14 to plasma membrane preparations was found to be dependent upon incubation temperature and duration. Maximal binding occurred after 35 min and remained constant until 120 min (data not shown). Although binding characteristics at 21°C (room temperature) and 35°C were similar, 20–30% higher specific bound counts were observed at the higher temperature. In view of these data, subsequent experiments were performed with a 60 min incubation period at 35°C.

A linear relationship was found between plasma membrane protein concentration and $^{125}$I-tyr-11-somatostatin binding over the protein range 10–200 µg membrane protein in a total reaction volume of 100 µl (0.1–2.0 mg ml$^{-1}$). Plasma membranes were routinely assayed at a protein concentration of 0.2–0.5 mg ml$^{-1}$.

Scatchard analysis of binding to rat cerebral cortex revealed a single class of specific high affinity receptors with a median (range) receptor affinity of 0.85 (0.55–0.99) nM and a Bmax of 0.22 (0.12–0.32 pmol mg$^{-1}$ protein (Figure 1). These findings are consistent with those reported previously (Srikant & Patel, 1981; Reubi & Maurer, 1986).

*Somatostatin binding in gastrointestinal tumours and normal mucosa*

Specific somatostatin binding sites were characterised in 23 out of 28 gastric cancers, 17 out of 23 colonic cancers, 21 out of 28 samples of gastric body mucosa and in 15 out of 23 samples of colonic mucosa. In the remaining tissue specimens there was insufficient displaceable somatostatin binding for Scatchard analysis.

In human gastric adenocarcinoma, 22 of 28 tumours exhibited specific low affinity binding of $^{125}$I-tyr-11-somatostatin-14, with an affinity of 170 (72–250) nM and a Bmax of 1.2 (0.7–5.7) pmol mg$^{-1}$ protein. A typical displacement curve with Scatchard plot is presented in Figure 2. In one additional patient, high affinity receptors were demonstrated with a Kd of 0.9 nM and Bmax of 0.23 pmol mg$^{-1}$ protein. Further detailed histochemical analysis of this tumour revealed that this was a typical gastric adenocarcinoma with no features to suggest a neuroendocrine origin. Specifically, staining for neurone-specific enolase, protein gene product 9.5 and chromogranin were negative.

**Figure 1** Displacement of $^{125}$I-tyr-11-somatostatin-14 from rat cerebral cortex membranes by somatostatin-14. Results shown are from a single representative experiment. $IC_{50}$ value was estimated to be 1.1 nM. Inset. Scatchard plot (Bound/Free against Bound, Bound as pmol mg$^{-1}$ protein, Free as nM) of the displacement data as calculated by LIGAND (26), with á Kd of 0.92 nM and a Bmax of 0.21 pmol mg$^{-1}$ protein.

**Figure 2** Displacement of $^{125}$I-tyr-11-somatostatin-14 from human gastric adenocarcinoma membranes by somatostatin-14. Results shown are from a single representative experiment. $IC_{50}$ value was estimated to be 158 nM. Inset. Scatchard plot as in Figure 1, revealing a site with a Kd of 165 nM and a Bmax of 6.1 pmol mg$^{-1}$ protein.

In colorectal adenocarcinoma, 17 of 23 of plasma membrane preparations exhibited low affinity, high capacity binding with a Kd of 140 (89–200) nM and Bmax of 1.8 (1.2–2.9) pmol mg$^{-1}$ protein.

Low affinity, high capacity binding was demonstrable in 21 of 28 gastric body mucosal plasma membrane preparations, including the sample from the patient whose tumour exhibited high affinity binding, with a Kd of 180 (81–270) nM and Bmax of 3.9 (1.2–6.6) pmol mg$^{-1}$ protein. These values are not significantly different from those found in the gastric carcinoma tissue.

In normal colonic mucosa, specific somatostatin binding of similar affinity and receptor density (Kd of 130 (71–200) nM; Bmax of 0.7 (0.3–3.5) pmol mg$^{-1}$ protein) to that in the stomach was demonstrated in 15 of 23 plasma membrane preparations. There were no statistically significant differences in receptor characteristics between normal and malignant colorectal samples.

*Relationship between binding characteristics and tumour site, stage and grade*

Details of the tumours' site, grade and stage are shown in Table I, along with the binding characteristics. There was no relationship between any of the tumour characteristics and somatostatin binding.

### Discussion

The current report represents the first systematic examination of somatostatin binding in solid neoplasms of the gastrointestinal tract. We have demonstrated high levels of specific binding for this peptide in membrane preparations from the majority of human colorectal and gastric cancers, and that these binding sites are also present in normal mucosa. The binding in both malignant tissue and uninvolved mucosa is normally low affinity, with a Kd usually in the micromolar range, in contrast to the high affinity binding site we demonstrate in rat cerebral cortex. One gastric tumour, however, in repeated determinations, consistently exhibited a high affinity binding site indicating that this tissue expresses a different class of receptor. Interestingly, the binding in the adjacent normal mucosa in this patient was of the normal, low affinity, type which we characteristically demonstrate in mucosa.

The demonstration of low affinity binding in normal gastric mucosa may seem at variance with reports of somatostatin binding in isolated parietal cell preparations (Sjodin *et al.*, 1990; Reyl-Desmars & Lewin, 1982), although species variations and differences in ligand (Conlon *et al.*, 1981) make comparisons difficult. However, it is possible that high

**Table I** Somatostatin binding in colorectal and gastric cancer tissue

| | | *n* + *ve* | *Kd (nM)* | *Bmax (pmol mg$^{-1}$ protein)* |
|---|---|---|---|---|
| *Colo-rectal Cancer* | Site | | | |
| | - Rectum | 8/12 | 112 (71–180) | 1.67 (1.02–2.78) |
| | - Sigmoid | 3/5 | 161 (10–181) | 0.49 (0.11–1.75) |
| | - (L) Colon | 0/1 | / | / |
| | - (R) Colon | 5/5 | 140 (97–200) | 2.99 (2.12–3.94) |
| | Stage | | | |
| | - A | 1/1 | 300 | 3.40 |
| | - B | 7/11 | 89 (53–200) | 1.72 (1.30–2.99) |
| | - C | 8/11 | 143 (104–171) | 1.48 (0.62–2.41) |
| | Grade | | | |
| | - Well | 5/8 | 161 (84–181) | 1.75 (0.49–2.99) |
| | - Mod | 9/12 | 113 (89–145) | 1.72 (1.30–2.69) |
| | - Poor | 2/3 | 163 (111–215) | 1.81 (0.74–2.87) |
| *Gastric Cancer* | Site | | | |
| | - Body | 10/11 | 78 (45–185) | 0.83 (0.25–7.43) |
| | - Antrum | 10/12 | 165 (78–169) | 1.04 (0.67–3.36) |
| | - Cardia | 5/5 | 265 (236–293) | 1.73 (1.23–5.11) |
| | Stage | | | |
| | - I | 1/2 | 89.2 | 0.28 |
| | - II | 6/6 | 78 (67–165) | 0.79 (0.23–1.04) |
| | - III | 17/19 | 168 (73–265) | 1.24 (0.77–6.11) |
| | - IV | 1/1 | 169 | 6.3 |
| | Grade | | | |
| | - Well | 1/3 | 265 | 5.11 |
| | - Mod | 17/7 | 165 (67–298) | 1.04 (0.23–6.23) |
| | - Poor | 17/18 | 162 (73–185) | 1.17 (0.67–5.71) |

The data from 23 patients with colorectal cancer and 28 with gastric cancer are demonstrated. 'n' refers to the number of samples in which there was sufficient displaceable binding to allow Scatchard analysis. The Kd (receptor affinity) and Bmax (total number of binding sites) are shown for each site, stage and grade of tumour.

affinity binding, which may be found in certain cell population in the mucosa, is not being detected by our technique which employs a whole tissue membrane preparation. It is of interest that Reubi and Maurer (1986) have demonstrated a low affinity receptor site in rat cerebral cortex, although this 'low affinity' site has a much higher affinity than that which we have identified.

In many receptor binding systems, it is assumed that functioning receptors require high ligand affinity such that optimum binding can occur at physiological hormone concentrations. That only one tumour of 51 assayed should express such high affinity receptor sites for somatostatin may seem disappointing since it implies that therapy based upon long-acting somatostatin analogues may be of benefit in a very small minority of patients. The incidence of high affinity receptors for other hormones, such as gastrin, in colonic carcinoma has been reported as 50–60% (Upp *et al*., 1989). Thus, gastrin antagonist therapy may be a reasonable therapeutic modality in receptor positive cases. However, the physiological and functional significance of such low affinity somatostatin binding is less easily evaluated.

One possible explanation for the function of a low affinity receptor is that it may be involved in local regulation, in an autocrine or paracrine fashion, as opposed to a true endocrine mechanism. Low circulating levels of somatostatin may be functionally insignificant whilst high local levels within the mucosa/tumour may be active. These findings are of importance if therapy based on the natural anti-proliferative effects of somatostatin is to be considered. Clearly, the native peptide cannot be used as a pharmacologically useful agent since its half life in the circulation is less than 3 min. However, current analogues of somatostatin, which are based upon a cyclic octapeptide sequence, have been found to have 100–200 times the suppressive effect on growth hormone release of native somatostatin with biological half lives of 3–6 h (Cai *et al*., 1986). Thus, used in pharmacological doses, appropriately designed analogues may be expected to have significant effects on low affinity receptors.

The results of our studies do not directly tell us whether somatostatin analogues will be of value in gastrointestinal cancer since firstly, the functional significance of the binding we have demonstrated is not clear and, secondly, somatostatin may exert growth effects independently of its interaction with membrane bound receptors on tumour cells. The direct, receptor mediated effects of somatostatin on cell growth appear to relate to the dephosphorylation of intracellular phosphoproteins (Reyl & Lewin, 1982). It is known that EGF stimulates cell growth by inducing phosphorylation of these residues and, as a corollary, somatostatin induced dephosphorylation has been suggested to correlate with inhibition of EGF stimulated cell growth in certain cancer cell lines (Liebow *et al*., 1989; Hierowski *et al*., 1985). Further studies, including examination of second messenger activation and affinity labelling may elucidate the therapeutic potential of the interaction between somatostatin and low affinity binding sites in human gastrointestinal cancer.

G.V.M. was supported by a grant from Glaxo Group Research Ltd.

### References

ADRIAN, T.E., BARNES, A.J., LONG, R.G., O'SHAUGHNESSY, D.J., BROWN, M.R., RIVIER, J., VALE, W., BLACKBURN, A.M. & BLOOM, S.R. (1981). The effect of somatostatin analogues on secretion of growth, pancreatic and gastrointestinal hormones in man. *J. Clin. Endocrinol. Metab.*, **53**, 675–681.

BLEIBERG, H. (1990). The future of adjuvant treatment in gastrointestinal cancer. *Eur. J. Surg. Oncol.*, **16**, 189–194.

BRADFORD, M.M. (1976). A rapid and sensitive method for the quantitation of microgram quantities of protein utilising the principle of protein dye binding. *Anal. Biochem.*, **72**, 248–254.

CAI, R.-Z., SZOKE, B., LU, D., FU, T., REDDING, T.W. & SCHALLY, A.V. (1986). Synthesis and biological activity of highly potent octapeptide analogs of somatostatin. *Proc. Natl Acad. Sci. USA*, **83**, 1896–1900.

CONLON, J.M., WHITTAKER, J., HAMMOND, V. & ALBERTI, K.G.M.M. (1981). Metabolism of somatostatin and its analogues by the liver. *Biochim. et Biophys. Acta*, **677**, 234–242.

CRILE, G. Jnr. (1957). The endocrine dependency of certain thyroid cancers and the danger that hypothyroidism may stimulate their growth. *Cancer*, **10**, 1119–1137.

CZERNIK, A. & PETRACK, B. (1983). Somatostatin receptor binding in rat cerebral cortex, characterisation using a non-reducible analog. *J. Biol. Chem.*, **258**, 5525–5530.

ENGSTROM, P.F., LAVIN, P.T., DOUGLASS, H.O. & BRUNNER, K.W. (1985). Postoperative adjuvant 5-Fluorouracil plus Methyl-CCNU therapy for gastric cancer patients. *Cancer*, **55**, 1868–1873.

FIELDING, J.W.L., FAGG, S.L., JONES, B.G., ELLIS, D., HOCKEY, M.S., MINAWA, A., BROOKES, V.S., CRAVEN, J.L., MASON, M.C., TIMOTHY, A., WATERHOUSE, J.A.H. & WRIGLEY, P.F.M. (1983). An interim report of a prospective, randomised, controlled study of adjuvant chemotherapy in operable gastric cancer: British Stomach Cancer Group. *World J. Surg.*, **7**, 390–399.

HIEROWSKI, M., LIEBOW, C., DU SAPIN, K. & SCHALLY, A.V. (1985). Stimulation by somatostatin of dephosphorylation of membrane proteins in pancreatic cancer MIAPaCa2 cell line. *FEBS Lett.*, **179**, 252–256.

HIGGINS, C. & HODGES, C.O. (1941). Studies on prostatic cancer. 1. The effect of castration, of oestrogen and of androgen injection on serum phosphatases in metastatic carcinoma of the prostate. *Cancer Res.*, **1**, 293–297.

HOROWITZ, K.G., MCGUIRE, W.L., PEARSON, O.H. & SEGALOFF, A. (1975). Predicting response to endocrine therapy in human breast cancer; a hypothesis. *Science*, **189**, 726–727.

HOWATSON, A.G. & CARTER, D.C. (1985). Pancreatic carcinogenesis-enhancement by cholecystokinin in the hamster-nitrosamine model. *Br. J. Cancer*, **51**, 107–114.

IKUYAMA, S., NAWATA, H., KATO, K., KARASHIMA, T., IBAYASHI, H. & NAKAGAKI, H. (1985). Specific somatostatin receptors on human pituitary adenoma cell membranes. *J. Clin. Endocrinol. Metab.*, **61**, 666–671.

KIRKEGAARD, P., OLSEN, P.S., NEXO, E., HOLST, J.J. & POULSEN, S.S. (1984). Effect of vasoactive intestinal polypeptide and somatostatin on secretion of epidermal growth factor and bicarbonate from Brunner's glands. *Gut*, **25**, 1225–1229.

LEHY, T., DUBRASQUET, M. & BONFILS, S. (1979). Effect of somatostatin on normal and gastrin stimulated cell proliferation in the gastric and intestinal mucosae of the rat. *Digestion*, **19**, 99–109.

LI, A.K.C., SCHATTENKERK, M.E., DEVRIES, J.E., FORD, W.D.A. & MALT, R.A. (1980). Submandibular siladenectomy retards dimethylhydrazine-induced colonic carcinogenesis. *Gastroenterology*, **78**, 1207.

LIEBOW, C., REILLY, C., SERRANO, M. & SCHALLY, A.V. (1989). Somatostatin analogues inhibit growth of pancreatic cancer by stimulating tyrosine phosphatase. *Proc. Natl Acad. Sci. USA*, **86**, 2003–2007.

MCMICHAEL, A.J. & POTTER, J.D. (1980). Reproduction, endogenous and exogenous sex hormones and colon cancer: A review. *J. Natl Cancer Inst.*, **65**, 1201–1207.

MORRIS, D.L., WATSON, S.A., HARRISON, J.D. & DURRANT, L. (1988). Somatostatin (SMS201995) reduces growth of human gastric cancer (MKN45) xenografts in nude mice. *Gut*, **30**, A1477.

MUNSON, P.J. & RODBARD, D. (1980). LIGAND: A versatile computerised approach for characterisation of ligand binding systems. *Anal. Biochem.*, **107**, 220–239.

OSEI, K.W., O'DORISIO, T.M. & ELLISON, E.C. (1985). Malignant insulinoma: effects of a somatostatin analog (compound 201995) on serum glucose, growth and gastroenteropancreatic hormones. *Ann. Intern. Med.*, **103**, 223–225.

RAKE, M.O., MALLINSON, C.N., COCKING, J.B., CWYNARSKI, M.T., FOX, C.A., WASS, V.J., DIFFEY, B.L. & JACKSON, G.A. (1979). Chemotherapy in advanced gastric cancer: a controlled, prospective, randomised multi-centre study. *Gut*, **20**, 797–801.

REUBI, J.C. & MAURER, R. (1986). Different ionic requirements for somatostatin receptor subpopulations in the brain. *Regulat. Pept.*, **14**, 301–311.

REUBI, J.C., LANG, W., MAURER, R., KOPER, J.W. & LAMBERTS, S.W.J. (1987*a*). Distribution and biochemical characterisation of somatostatin receptors in tumours of the human central nervous system. *Cancer Res.*, **47**, 5758–5764.

REUBI, J.C., HACKI, W.H. & LAMBERTS, S.W.J. (1987*b*). Hormone-producing gastrointestinal tumours contain high density of somatostatin receptors. *J. Clin. Endocrin. Metab.*, **65**, 1127–1134.

REUBI, J.C., WASER, B., SHEPPARD, M. & MACAULAY, V. (1990). Somatostatin receptors are present in small-cell but not in non-small-cell primary lung cancers. *Int. J. Cancer*, **45**, 269–274.

REYL-DESMARS, F. & LEWIN, M.J.M. (1982). Evidence for an intracellular somatostatin receptor in pancreas: A comparative study with reference to gastric mucosa. *Biochem. Biophys. Res. Com.*, **109**, 1324–1331.

REYL, F.J. & LEWIN, M.J.M. (1982). Intracellular receptor for somatostatin in gastric mucosal cells: Decomposition and reconstitution of somatostatin-stimulated phosphoprotein phosphatases. *Proc. Natl Acad. Sci. USA*, **79**, 978–982.

SINGH, P., LE, S., TOWNSEND, C.M., BEAUCHAMP, R.D., LARINDJANI, A., & THOMPSON, J.C. (1986). A long acting somatostatin analog and proglumide inhibit the trophic and gastrin receptor regulatory effects of pentagastrin on mouse colon cancer cells *in vivo*. *Gastroenterology*, **90**, 1636.

SIRINEK, K.R., LEVINE, B.A. & MOYER, M.P. (1985). Pentagastrin stimulates in vitro growth of normal and malignant human colon epithelial cells. *Am. J. Surg.*, **149**, 35–39.

SJODIN, L., ENGLUND, L.J. & MARDH, S. (1990). Binding of cholecystokinin and somatostatin to isolated porcine gastric mucosal cells and effect on aminopyrine uptake. *Acta Physiol. Scand.*, **138**, 369–376.

SRIKANT, C.B. & PATEL, Y.C. (1981). Somatostatin receptors: identification and characterisation in rat brain membranes. *Proc. Natl Acad. Sci. USA*, **78**, 3930–3934.

TOWNSEND, C.M., SINGH, P. & THOMPSON, J.C. (1986). Gastrointestinal hormones and gastrointestinal and pancreatic carcinomas. *Gastroenterology*, **91**, 1002–1006.

UPP, J.R. Jr, SINGH, P., TOWNSEND, C.M. Jr & THOMPSON, J.C. (1989). Clinical significance of gastrin receptors in human colon cancers. *Cancer Res.*, **49**, 488–492.

WATSON, S.A., DURRANT, L.G. & MORRIS, D.L. (1988). Growth-promoting action of gastrin on human colonic and gastric tumor cells cultured *in vitro*. *Br. J. Surg.*, **75**, 342–345.


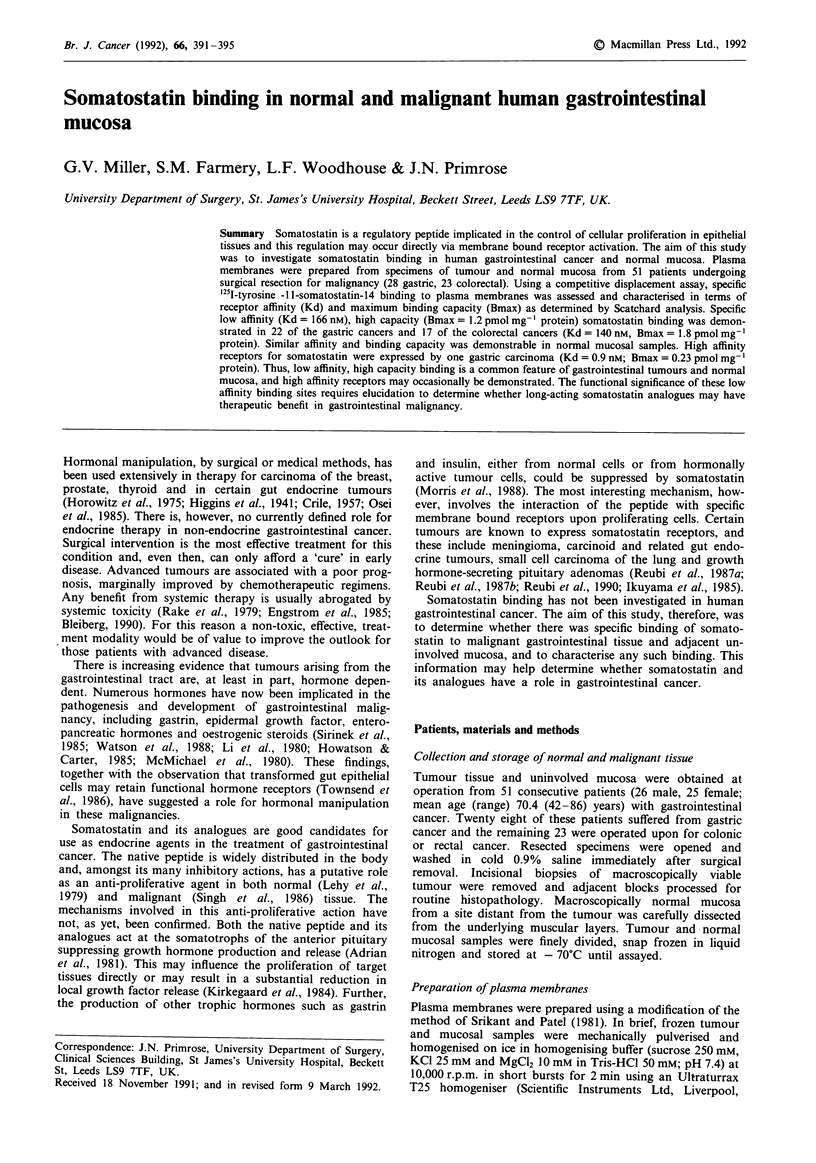

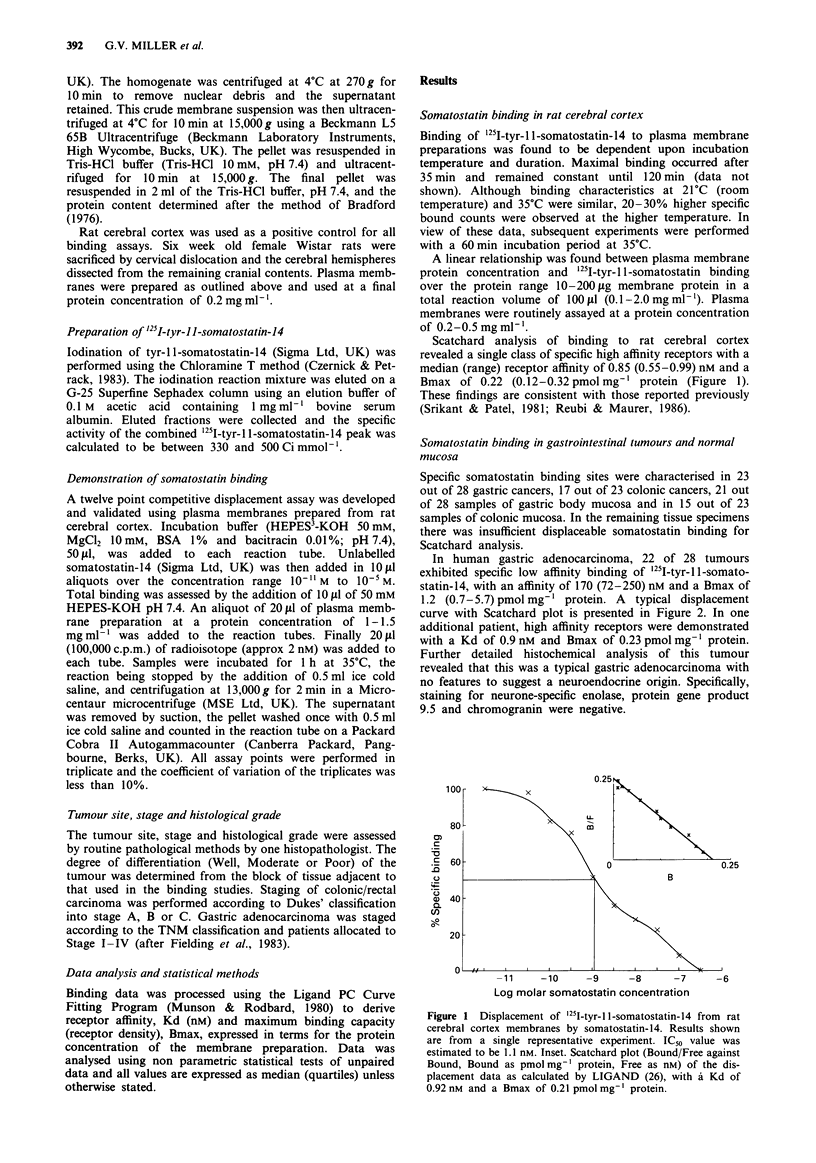

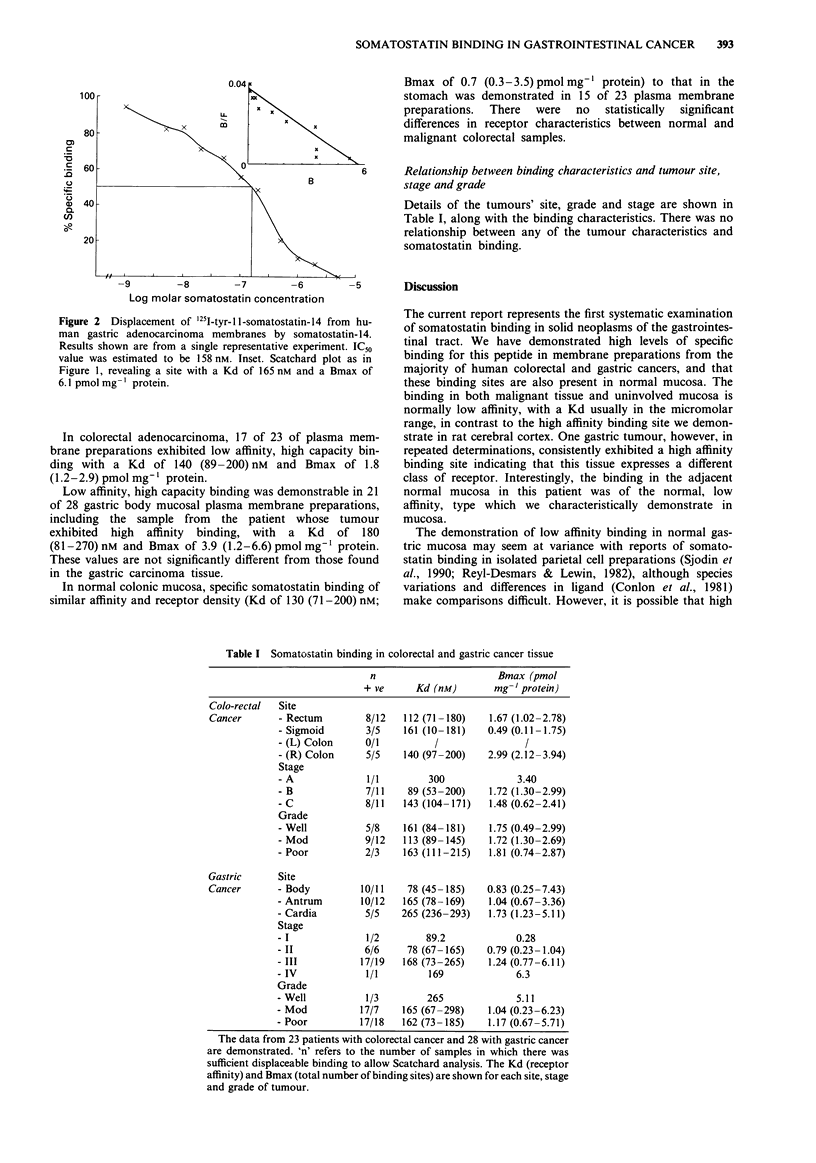

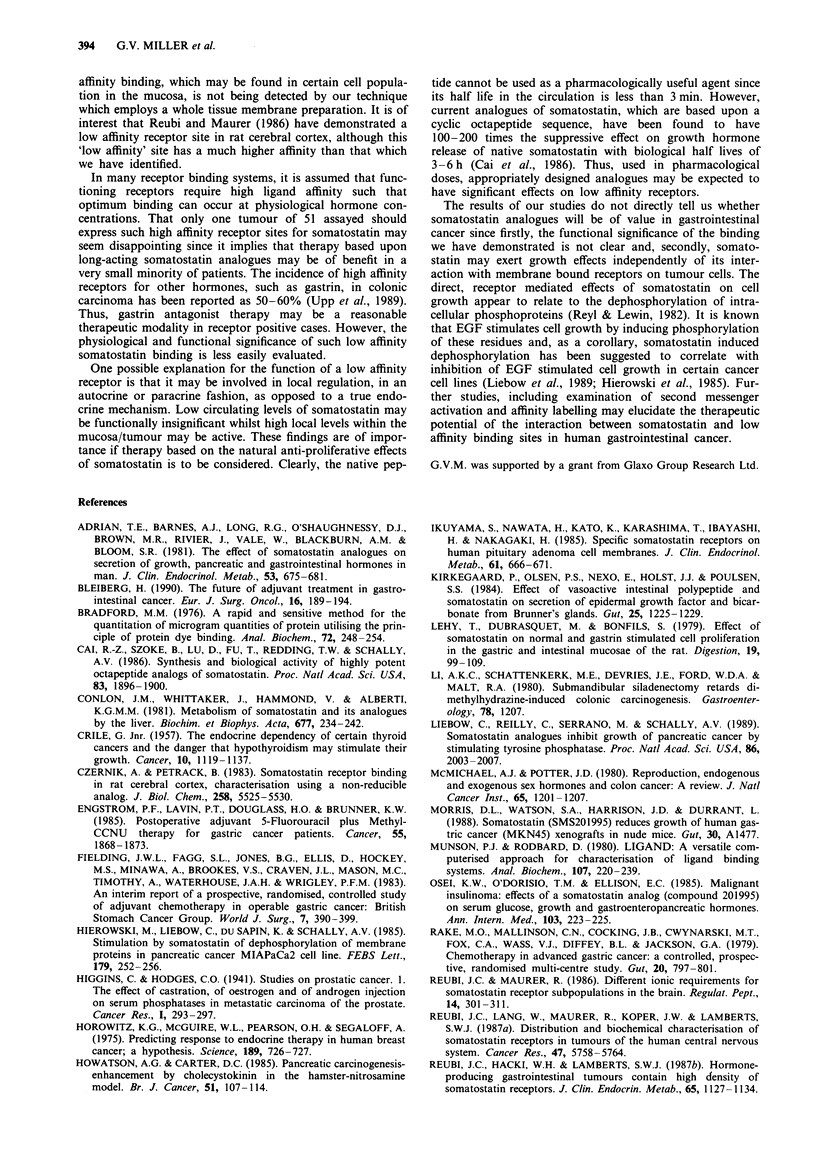

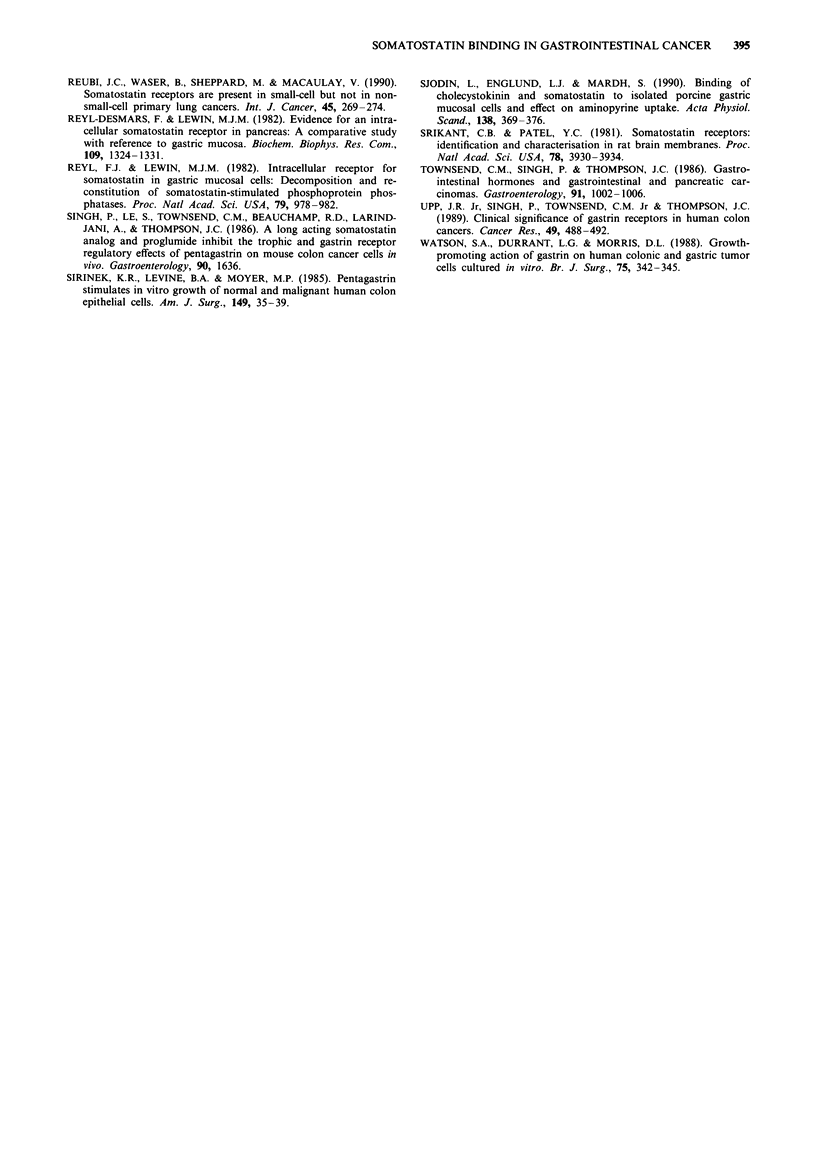


## References

[OCR_00486] Adrian T. E., Barnes A. J., Long R. G., O'Shaughnessy D. J., Brown M. R., Rivier J., Vale W., Blackburn A. M., Bloom S. R. (1981). The effect of somatostatin analogs on secretion of growth, pancreatic, and gastrointestinal hormones in man.. J Clin Endocrinol Metab.

[OCR_00493] Bleiberg H. (1990). The future of adjuvant treatment in gastrointestinal cancer.. Eur J Surg Oncol.

[OCR_00497] Bradford M. M. (1976). A rapid and sensitive method for the quantitation of microgram quantities of protein utilizing the principle of protein-dye binding.. Anal Biochem.

[OCR_00513] CRILE G. (1957). The endocrine dependency of certain thyroid cancers and the danger that hypothyroidism may stimulate their growth.. Cancer.

[OCR_00502] Cai R. Z., Szoke B., Lu R., Fu D., Redding T. W., Schally A. V. (1986). Synthesis and biological activity of highly potent octapeptide analogs of somatostatin.. Proc Natl Acad Sci U S A.

[OCR_00508] Conlon J. M., Whittaker J., Hammond V., Alberti K. G. (1981). Metabolism of somatostatin and its analogues by the liver.. Biochim Biophys Acta.

[OCR_00518] Czernik A. J., Petrack B. (1983). Somatostatin receptor binding in rat cerebral cortex. Characterization using a nonreducible somatostatin analog.. J Biol Chem.

[OCR_00523] Engstrom P. F., Lavin P. T., Douglass H. O., Brunner K. W. (1985). Postoperative adjuvant 5-fluorouracil plus methyl-CCNU therapy for gastric cancer patients. Eastern Cooperative Oncology Group study (EST 3275).. Cancer.

[OCR_00529] Fielding J. W., Fagg S. L., Jones B. G., Ellis D., Hockey M. S., Minawa A., Brookes V. S., Craven J. L., Mason M. C., Timothy A. (1983). An interim report of a prospective, randomized, controlled study of adjuvant chemotherapy in operable gastric cancer: British Stomach Cancer Group.. World J Surg.

[OCR_00537] Hierowski M. T., Liebow C., du Sapin K., Schally A. V. (1985). Stimulation by somatostatin of dephosphorylation of membrane proteins in pancreatic cancer MIA PaCa-2 cell line.. FEBS Lett.

[OCR_00549] Horwitz K. B., McGuire W. L. (1975). Predicting response to endocrine therapy in human breast cancer: a hypothesis.. Science.

[OCR_00554] Howatson A. G., Carter D. C. (1985). Pancreatic carcinogenesis-enhancement by cholecystokinin in the hamster-nitrosamine model.. Br J Cancer.

[OCR_00559] Ikuyama S., Nawata H., Kato K., Karashima T., Ibayashi H., Nakagaki H. (1985). Specific somatostatin receptors on human pituitary adenoma cell membranes.. J Clin Endocrinol Metab.

[OCR_00565] Kirkegaard P., Olsen P. S., Nexø E., Holst J. J., Poulsen S. S. (1984). Effect of vasoactive intestinal polypeptide and somatostatin on secretion of epidermal growth factor and bicarbonate from Brunner's glands.. Gut.

[OCR_00571] Lehy T., Dubrasquet M., Bonfils S. (1979). Effect of somatostatin on normal and gastric-stimulated cell proliferation in the gastric and intestinal mucosae of the rat.. Digestion.

[OCR_00583] Liebow C., Reilly C., Serrano M., Schally A. V. (1989). Somatostatin analogues inhibit growth of pancreatic cancer by stimulating tyrosine phosphatase.. Proc Natl Acad Sci U S A.

[OCR_00589] McMichael A. J., Potter J. D. (1980). Reproduction, endogenous and exogenous sex hormones, and colon cancer: a review and hypothesis.. J Natl Cancer Inst.

[OCR_00598] Munson P. J., Rodbard D. (1980). Ligand: a versatile computerized approach for characterization of ligand-binding systems.. Anal Biochem.

[OCR_00603] Osei K., O'Dorisio T. M. (1985). Malignant insulinoma: effects of a somatostatin analog (compound 201-995) on serum glucose, growth, and gastro-entero-pancreatic hormones.. Ann Intern Med.

[OCR_00609] Rake M. O., Mallinson C. N., Cocking J. B., Cwynarski M. T., Fox C. A., Wass V. J., Diffey B. L., Jackson G. A. (1979). Chemotherapy in advanced gastric cancer: a controlled, prospective, randomised multi-centre study.. Gut.

[OCR_00626] Reubi J. C., Häcki W. H., Lamberts S. W. (1987). Hormone-producing gastrointestinal tumors contain a high density of somatostatin receptors.. J Clin Endocrinol Metab.

[OCR_00620] Reubi J. C., Lang W., Maurer R., Koper J. W., Lamberts S. W. (1987). Distribution and biochemical characterization of somatostatin receptors in tumors of the human central nervous system.. Cancer Res.

[OCR_00615] Reubi J. C., Maurer R. (1986). Different ionic requirements for somatostatin receptor subpopulations in the brain.. Regul Pept.

[OCR_00633] Reubi J. C., Waser B., Sheppard M., Macaulay V. (1990). Somatostatin receptors are present in small-cell but not in non-small-cell primary lung carcinomas: relationship to EGF-receptors.. Int J Cancer.

[OCR_00638] Reyl-Desmars F., Lewin M. J. (1982). Evidence for an intracellular somatostatin receptor in pancreas: a comparative study with reference to gastric mucosa.. Biochem Biophys Res Commun.

[OCR_00644] Reyl F. J., Lewin M. J. (1982). Intracellular receptor for somatostatin in gastric mucosal cells: decomposition and reconstitution of somatostatin-stimulated phosphoprotein phosphatases.. Proc Natl Acad Sci U S A.

[OCR_00657] Sirinek K. R., Levine B. A., Moyer M. P. (1985). Pentagastrin stimulates in vitro growth of normal and malignant human colon epithelial cells.. Am J Surg.

[OCR_00662] Sjödin L., Englund L. J., Mårdh S. (1990). Binding of cholecystokinin and somatostatin to isolated porcine gastric mucosal cells and effects on aminopyrine uptake.. Acta Physiol Scand.

[OCR_00668] Srikant C. B., Patel Y. C. (1981). Somatostatin receptors: identification and characterization in rat brain membranes.. Proc Natl Acad Sci U S A.

[OCR_00673] Townsend C. M., Singh P., Thompson J. C. (1986). Gastrointestinal hormones and gastrointestinal and pancreatic carcinomas.. Gastroenterology.

[OCR_00678] Upp J. R., Singh P., Townsend C. M., Thompson J. C. (1989). Clinical significance of gastrin receptors in human colon cancers.. Cancer Res.

[OCR_00683] Watson S. A., Durrant L. G., Morris D. L. (1988). Growth-promoting action of gastrin on human colonic and gastric tumour cells cultured in vitro.. Br J Surg.

